# Abdominal Section for Fecal Impaction

**Published:** 1905-11

**Authors:** John M. Neel

**Affiliations:** Bonham, Texas


For Texas Medical Journal.

### Abdominal Section for Fecal Impaction.

BY JOHN M. NEEL, M. D., BONHAM, TEXAS.

I am aware of the fact that in almost all cases of fecal impaction, the treatment is medical rather than surgical. These cases are most common in the adult female who has a history of dyspepsia and constipation; however, it is sometimes produced by trauma. The cecum transverse colon and sigmoid flexure are the common seat of obstruction. It is produced by a weakened peristaltic action which permits an accumulation of fecal matter below the paralyzed portion of the intestines. The bowel becomes contracted and increases the opposition to the advance of the intestinal contents, which produces not only impaction but also paralysis of the bowels. The diagnosis of the variety of lesion is of great clinical importance, but for all practical purposes, it is necessary to know only that some lesion exists demanding interference.

I report a case of Miss J. M., age 20 years. I first saw her at Bells, Texas, the 8th day of August of last year. She had been constipated for four weeks, having had only a few small actions from the lower bowels. Her abdomen was swollen and tender, and you could feel a tumor over the hepatic flexure of the colon. She had some appetite, and rested very well at night; kidneys acted fairly well; temperature normal.

I put her under an anesthetic, and after satisfying myself that it was a fecal accumulation, I tried to break it up, but failed.

Dr. B. had given her everything in the way of medicine, also colonic irrigations of both water and hot oil, with hips elevated, with no effect whatever. Dr. R. was called in consultation. They continued the irrigation with heroic doses of castor oil per orum for several days without any result. After all mild measures had failed, I was satisfied the bowel was paralyzed, and advised an exploratory incision, which was readily accepted. She was brought to Bonham and placed in the Allen Memorial Hospital. After careful preparation we opened the abdomen, and found the transverse colon to be the seat of the impaction. There were two strictures in the colon, and consequently two points of impaction. The blood vessels were congested and the bowel, especially between the impactions, was paralyzed and very dark, almost like the condition we find in strangulated hernia. The bowels were brought out on hot towels and the impactions carefully broken up, and stripping the contents of the bowels down the line, I closed the incision and dressed in the usual way. The second day I placed her on her right side and flushed the colon, and there was a great quantity of hard balls passed for several days. She continued to pass hardened feces and at the same time all of her symptoms began to clear up, and she returned to Bells the twentieth day. She required very little after-treatment. At this writing she is in perfect health.

